# Reactivation of BCG vaccination SCAR after influenza vaccination: a case report

**DOI:** 10.1093/omcr/omaf122

**Published:** 2025-07-27

**Authors:** Augustinas Stasiūnas, Jurgita Stasiūnienė, Enrika Didžiulienė

**Affiliations:** Faculty of Medicine, Vilnius University, M. K. Čiurlionio str. 21, Vilnius 03101, Vilnius County, Lithuania; Department of Pathology, Forensic Medicine, Institute of Biomedical Sciences of the Faculty of Medicine of Vilnius University, M. K. Čiurlionio str. 21/27, Vilnius 03101, Vilnius County, Lithuania; Department of Family Medicine, InMedica Medical Clinic, L. Asanavičiūtės g. 20, Vilnius 04303, Vilnius County, Lithuania

**Keywords:** bacillus Calmette-Guerin vaccine, BCG, influenza vaccine, BCG scar reactivation, erythema

## Abstract

The Bacillus Calmette-Guerin (BCG) vaccine, a live attenuated vaccine derived from *Mycobacterium bovis*, is widely used for tuberculosis prevention and has been linked to various immunological responses beyond its intended purpose. A 23-years-old healthy and allergy-free man was vaccinated for the current year's influenza on his left arm. Two days after inoculation, the patient’s BCG scar on his left arm was erythematous, while the influenza vaccination site (located 3 cm from the BCG scar) remained unchanged. A possible ipsilateral relationship between the BCG scar and the influenza vaccine site is suggested. BCG vaccination influences the increase in TNF-α and IL-6 production following influenza vaccination. In BCG-vaccinated subjects, hemagglutinin-inhibition antibody responses against the A(H1N1)pdm09 vaccine strain is markedly enhanced, with a trend toward more-rapid seroconversion. Understanding this BCG and influenza vaccines interaction is crucial for healthcare providers to differentiate between benign post-vaccination reactions and those that may require further clinical evaluation.

## Introduction

Bacillus Calmette-Guerin (BCG) vaccine is a live attenuated vaccine derived from *Mycobacterium bovis*. It is one of the most widely used vaccines in the world, and is primarily used for the prevention of tuberculosis [1–3]. Also, it has been associated with various immunological responses beyond its intended purpose. BCG vaccine also offers protection against non-tuberculous mycobacterial infections like leprosy and Buruli ulcer, nonspecific protective effects against protozoan infections, including tegumentary leishmaniasis and malaria. It is also used in the treatment of superficial carcinoma of the bladder [2–4]. There are also reports of the reactivation of a BCG vaccination scar following measles infection, as well as COVID-19 and influenza vaccinations [5–13]. The reactivation of the BCG scar, characterized by erythema and/or crust formation, is one of the pathognomonic features of Kawasaki disease and occurs in approximately 30-50% of Kawasaki disease patients [4, 14–17]. Here, we present a case report of a 23-years old man who presented with erythema at the BCG inoculation site following influenza vaccination. This article is important for understanding mechanisms of BCG scar reactivation after influenza vaccination, the clinical implications of this response, and the potential impact on vaccination strategies in populations with prior BCG immunization. Understanding this interaction is crucial for healthcare providers to differentiate between benign post-vaccination reactions and those that may require further clinical evaluation.

## A case report

**Figure 1:**
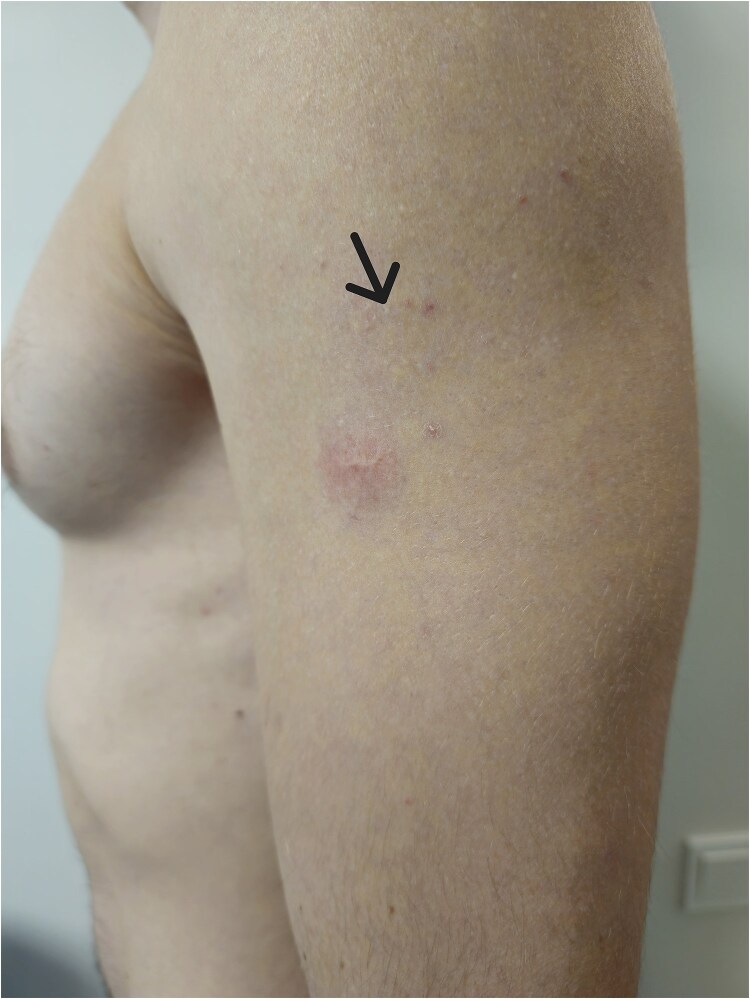
The erythema around the BCG scar. An arrow indicates the influenza vaccination site.

A 23-years-old healthy and allergy-free man was vaccinated for the current year's influenza on his left arm. Two days after inoculation, the patient presented with erythema on the deltoid muscle of the left arm at the site of a previous BCG vaccine scar. The BCG scar was erythematous, while the influenza vaccination site (located 3 cm from the BCG scar) remained unchanged. The erythema around the BCG scar measured 2 cm in diameter on the second day after the influenza vaccination (Fig. 1). By the fourth day, the erythema started to diminish and disappeared by day seven without any treatment. The patient reported receiving the influenza vaccine every two years and had not experienced any side effects previously. He was born in Lithuania and received the BCG vaccine on his left arm at two days of age. Our patient received a quadrivalent influenza vaccine containing the following strains: A/Victoria/4897/2022 (H1N1)pdm09-like strain (A/Victoria/4897/2022 IVR-238), A/Darwin/9/2021 (H3N2)-like strain (A/Darwin/9/2021 IVR-228), B/Austria/1359417/2021-like strain (B/Michigan/01/2021, wild-type), B/Phuket/3073/2013-like strain (B/Phuket/3073/2013, wild-type).

## Discussion

Our case report discusses an adult who experienced BCG scar reactivation following influenza vaccination. The erythema resolved within 7 days without treatment. Previous cases have been observed in children. In two of these cases, BCG scar induration and erythema lasted for 48-72 h and also disappeared without treatment [12, 13]. In another case, a day after vaccination, swelling, erosion, and blister formation were observed at the BCG scar site, which resolved 6 days post-vaccination following treatment that included an intravenous anti-allergic drug, topical corticosteroid, and antibiotic ointment [11]. More similar cases were observed after COVID-19 vaccination, where there was no pain or redness at the injection site, but these symptoms were observed exclusively at the BCG wheal. The symptoms following both the first and second doses resolved spontaneously without intervention after administration of both viral vector and mRNA vaccines [6, 8–10]. Our patient had also previously received three doses of the Covid-19 mRNA vaccine, but did not experience any BCG scar reactions. Some articles suggest a possible ipsilateral relationship between the BCG scar and the influenza or COVID-19 vaccine site [8, 11]. In other reports, no link was found between the side of the body where the COVID-19 or influenza vaccine was administered and the side of the body where the BCG scar became reactivated. However, BCG scar reactivation was also observed on the ipsilateral side [6, 12]. In our case, the influenza vaccine was administered in the same arm as the BCG scar. In one study, the reactivation of the BCG scar was observed on the contralateral side. However, during that study, the patients were revaccinated with the BCG vaccine and received the Covid-19 vaccine 6-8 months later, after which two individuals experienced reactivation of the new BCG scar. Both individuals had been vaccinated with BCG during childhood, and no reactivation was noted in the older scars. No treatment was needed or provided [7].

BCG vaccination is associated with enhanced cytokine responses after influenza, and also partially associated after SARS-CoV-2 stimulation. BCG vaccination influences the increase in TNF-α and IL-6 production induced by the influenza vaccination [3, 18]. Also, in animal models, a significant adjuvant effect of BCG cell wall skeleton (BCG-CWS) on the immune response to influenza vaccination has been observed, including decreased viral loads. BCG-CWS has a stimulating effect on antigen-presenting cells, inducing inflammatory cytokines through Toll-like receptors 2 and 4 signaling pathways. BCG-CWS exhibits multifunctional effects including protective humoral and cellular immune responses, control of excessive pathological inflammation, and Th1 cell proliferation upon influenza challenge [19, 20]. Also in BCG-vaccinated subjects, hemagglutinin-inhibition antibody responses against the A(H1N1)pdm09 vaccine strain is markedly enhanced, and there is a trend toward more-rapid seroconversion [3, 21]. It has also been observed in animal models that not only is BCG used to enhance immunization against the influenza virus, but influenza virus vectors can also induce a specific Th1 immune response against *Mycobacterium tuberculosis* [22].

One of the mechanisms proposed in the literature underlying BCG scar reactivation involves the mycobacterial heat shock protein (HSP)65 [10]. The amino acid sequences of mycobacterial HSP65 and those of the COVID-19 S (spike) and N (nucleocapsid) proteins are very similar. Both the COVID-19 S and N proteins support the possibility of a cross-immune reaction between BCG HSP65 and COVID-19 [23]. Furthermore, the BCG vaccine contains similar 9-amino acid sequences to those found in COVID-19; four sets of these similar peptides have been shown to have weak to high binding affinity for common HLA class I molecules. Thus, the BCG vaccine has the potential to generate cross-reactive T cells against COVID-19 [24].

Regarding BCG scar reactivation after influenza vaccination, the literature primarily discusses the nonspecific effects of BCG vaccination related to the enhanced function of myeloid antigen-presenting cells. It has been reported that BCG vaccination prior to influenza vaccination results in a more pronounced and accelerated induction of functional antibody responses against the 2009 pandemic influenza A(H1N1) vaccine strain [21]. Additionally, after influenza vaccination, increases in IL-6 and TNF-α are observed [3, 18]. Positive correlations have been found between serum levels of HSP70 and the inflammatory markers IL-6 and TNF-α, and a homology between HSP70 and mycobacterial antigens has been demonstrated [5, 25]. HSP70 enhances the cross-presentation of exogenous antigens on MHC class I, resulting in better antigen-specific T-cell stimulation [26]. When cytokines or increased levels of HSP70 are present, resident memory T cells (TRM) in the BCG scar can become reactivated. TRM cells rapidly activate local immune cells to mount a response that protects against infections by unrelated pathogens [27, 28]. The potent inflammatory effects of TRM reactivation may have pathological consequences, potentially contributing to the exacerbation of tissue-specific autoimmune or inflammatory diseases [28]. However, many studies report that BCG scar reactivation is typically benign and self-limiting, with no significant long-term adverse effects [6, 8–10, 12, 13]. Thus, BCG scar reactivation could be caused by the nonspecific immunological effects induced by the BCG vaccine and by the influenza vaccine–induced increase in HSP70 that is homologous to mycobacterial antigens.

The limitation of this case is that no laboratory tests were conducted to determine the pathogenesis causing the symptoms. However, this case could strengthen the idea proposed by other authors that the BCG vaccine reactivation response occurs when the influenza vaccine is administered in the ipsilateral arm. Understanding this could help clinicians reduce unwanted reactions to the influenza vaccine in individuals previously vaccinated with BCG and guide them in responding to such bodily reactions.

## References

[1] World Health Organization (WHO) . BCG vaccine. Geneva, Switzerland: World Health Organisation. https://www.who.int/teams/health-product-policy-and-standards/standards-and-specifications/norms-and-standards/vaccines-quality/bcg. Accessed October 10, 2024.

[2] Okafor CN, Rewane A, Momodu II. Bacillus Calmette Guerin. Treasure Island (FL): StatPearls Publishing; 2024. http://www.ncbi.nlm.nih.gov/books/NBK538185/. Accessed October 10, 2024. 30844212

[3] Parmar K, Siddiqui A, Nugent K. Bacillus Calmette-Guerin vaccine and nonspecific immunity. Am J Med Sci 2021;361:683–9. 10.1016/j.amjms.2021.03.003 33705721 PMC7938189

[4] Yamazaki-Nakashimada MA, Unzueta A, Berenise Gámez-González L. et al. BCG: a vaccine with multiple faces. Hum Vaccin Immunother 2020;16:1841–50. 10.1080/21645515.2019.1706930 31995448 PMC7482865

[5] Muthuvelu S, Lim KSC, Huang LY. et al. Measles infection causing bacillus Calmette-Guérin reactivation: a case report. BMC Pediatr 2019;19:251. 10.1186/s12887-019-1635-z 31340782 PMC6652017

[6] Tao J, Rosenfeld D, Hsu J. et al. Reactivation of a BCG vaccination scar following the first dose of the Moderna COVID-19 vaccine. Cutis. 2022;109:148–9. 10.12788/cutis.0470 35659140

[7] Mohamed L, Madsen AMR, Schaltz-Buchholzer F. et al. Reactivation of BCG vaccination scars after vaccination with mRNA-Covid-vaccines: two case reports. BMC Infect Dis 2021;21:1264. 10.1186/s12879-021-06949-0 34930152 PMC8685493

[8] Prasad BNS . Bacillus Calmette–Guérin scar inflammation after COVID vaccination. Indian J Rheumatol 2022;17:170–3. 10.4103/injr.injr_194_21

[9] Hung TK, Leung D, Duque JSR. et al. Bacillus Calmette-Guérin scar erythema in a 14-year-old girl post-BNT162b2 vaccination. Pediatr Int 2022;64:e15090. 10.1111/ped.15090 35175660 PMC9306499

[10] van Balveren L, van Puijenbroek EP, Davidson L. et al. A case series of bacillus Calmette-Guérin scar reactivation after administration of both mRNA and viral vector COVID-19 vaccines. Br J Clin Pharmacol 2023;89:2113–21. 10.1111/bcp.15678 36717367

[11] Kondo M, Goto H, Yamamoto S. First case of redness and erosion at bacillus Calmette–Guérin inoculation site after vaccination against influenza. J Dermatol 2016;43:1229–31. 10.1111/1346-8138.13365 27028876

[12] Chavarri-Guerra Y, Soto-Perez-de-Celis E. Erythema at the bacillus Calmette-Guerin scar after influenza vaccination. Rev Soc Bras Med Trop 2019;53:e20190390. 10.1590/0037-8682-0390-2019 31859956 PMC7083363

[13] das NF MT, de M-PMI, Costa-Carvalho BT. et al. Clinical management of localized BCG adverse events in children. Rev Inst Med Trop Sao Paulo 2016;58:84. 10.1590/S1678-9946201658084 27828625 PMC5096638

[14] Yang MC, Wu KL, Huang CN. et al. Kawasaki disease in children with bacillus Calmette-Guérin scar reactivity: focus on coronary outcomes. J Formos Med Assoc 2023;122:1001–7. 10.1016/j.jfma.2023.04.016 37142476

[15] Rezai MS, Shahmohammadi S. Erythema at BCG inoculation site in Kawasaki disease patients. Mater Sociomed 2014;26:256–60. 10.5455/msm.2014.26.256-260 25395889 PMC4214810

[16] Diniz LMO, Castanheira RG, Giampietro YG. et al. Diagnostic value of the reaction at the bacillus calmette-guérin vaccination site in Kawasaki disease. Rev Paul Pediatr 2021;39:e2019338. 10.1590/1984-0462/2021/39/2019338 32876305 PMC7457469

[17] Tsuboya N, Makino H, Mitani Y. et al. Erythema and induration of bacillus Calmette-Guérin scar associated with multisystem inflammatory syndrome in children in Japan: a case report. Front Pediatr 2022;10:849473. 10.3389/fped.2022.849473 35359902 PMC8963203

[18] Moorlag SJCFM, Taks E, ten Doesschate T. et al. Efficacy of BCG vaccination against respiratory tract infections in older adults during the coronavirus disease 2019 pandemic. Clin Infect Dis 2022;75:e938–46. 10.1093/cid/ciac182 35247264 PMC8903481

[19] Bhatnagar N, Kim KH, Subbiah J. et al. Comparison of the effects of different potent adjuvants on enhancing the immunogenicity and cross-protection by influenza virus vaccination in young and aged mice. Antivir Res 2022;197:105229. 10.1016/j.antiviral.2021.105229 34933043 PMC8801234

[20] Kim KH, Lee YT, Park Y. et al. BCG Cell Wall skeleton As a vaccine adjuvant protects both infant and old-aged mice from influenza virus infection. Biomedicines. 2021;9:516. 10.3390/biomedicines9050516 34063125 PMC8148143

[21] Leentjens J, Kox M, Stokman R. et al. BCG vaccination enhances the immunogenicity of subsequent influenza vaccination in healthy volunteers: a randomized. Placebo-Controlled Pilot Study J Infect Dis 2015;212:1930–8. 10.1093/infdis/jiv332 26071565

[22] Sereinig S, Stukova M, Zabolotnyh N. et al. Influenza virus NS vectors expressing the mycobacterium tuberculosis ESAT-6 protein induce CD4+ Th1 immune response and protect animals against tuberculosis challenge. Clin Vaccine Immunol 2006;13:898–904. 10.1128/CVI.00056-06 16893990 PMC1539114

[23] Finotti P . Sequence similarity of HSP65 of Mycobacterium bovis BCG with SARS-CoV-2 spike and nuclear proteins: may it predict an antigen-dependent immune protection of BCG against COVID-19? Cell Stress Chaperones 2022;27:37–43. 10.1007/s12192-021-01244-y 34755305 PMC8577642

[24] Tomita Y, Sato R, Ikeda T. et al. BCG vaccine may generate cross-reactive T cells against SARS-CoV-2: In silico analyses and a hypothesis. Vaccine. 2020;38:6352–6. 10.1016/j.vaccine.2020.08.045 32863070 PMC7440160

[25] Njemini R, Lambert M, Demanet C. et al. Elevated serum heat-shock protein 70 levels in patients with acute infection: use of an optimized enzyme-linked immunosorbent assay. Scand J Immunol 2003;58:664–9. 10.1111/j.1365-3083.2003.01341.x 14636423

[26] McNulty S, Colaco CA, Blandford LE. et al. Heat-shock proteins as dendritic cell-targeting vaccines--getting warmer. Immunology. 2013;139:407–15. 10.1111/imm.12104 23551234 PMC3719058

[27] Ariotti S, Hogenbirk MA, Dijkgraaf FE. et al. T cell memory. Skin-resident memory CD8^+^ T cells trigger a state of tissue-wide pathogen alert. Science. 2014;346:101–5. 10.1126/science.1254803 25278612

[28] Schenkel JM, Fraser KA, Beura LK. et al. Resident memory CD8 T cells trigger protective innate and adaptive immune responses. Science. 2014;346:98–101. 10.1126/science.1254536 25170049 PMC4449618

